# Multilevel social factors and NICU quality of care in California

**DOI:** 10.1038/s41372-020-0647-8

**Published:** 2020-03-10

**Authors:** Amy M. Padula, Salma Shariff-Marco, Juan Yang, Jennifer Jain, Jessica Liu, Shannon M. Conroy, Suzan L. Carmichael, Scarlett L. Gomez, Ciaran Phibbs, John Oehlert, Jeffrey B. Gould, Jochen Profit

**Affiliations:** 1 Department of Obstetrics, Gynecology and Reproductive Sciences, University of California, San Francisco, San Francisco, CA USA; 2 Department of Epidemiology and Biostatistics, University of California, San Francisco, San Francisco, CA USA; 3 Helen Diller Family Comprehensive Cancer Center, University of California, San Francisco, San Francisco, CA USA; 4 Department of Pediatrics, Stanford University School of Medicine, Palo Alto, CA USA; 5 Health Economics Resource Center, VA Palo Alto Health Care System, Menlo Park, CA USA

**Keywords:** Paediatrics, Epidemiology

## Abstract

**Objective:**

Our objective was to incorporate social and built environment factors into a compendium of multilevel factors among a cohort of very low birth weight infants to understand their contributions to inequities in NICU quality of care and support providers and NICUs in addressing these inequities via development of a health equity dashboard.

**Study design:**

We examined bivariate associations between NICU patient pool and NICU catchment area characteristics and NICU quality of care with data from a cohort of 15,901 infants from 119 NICUs in California, born 2008–2011.

**Result:**

NICUs with higher proportion of minority racial/ethnic patients and lower SES patients had lower quality scores. NICUs with catchment areas of lower SES, higher composition of minority residents, and more household crowding had lower quality scores.

**Conclusion:**

Multilevel social factors impact quality of care in the NICU. Their incorporation into a health equity dashboard can inform providers of their patients’ potential resource needs.

## Introduction

Persistent racial/ethnic inequities in adverse birth outcomes are the focus of many reports, though little progress has been made to improve equity in care and outcomes. Inequities often increase with severity of outcomes; for example, Black infants are at twofold risk of low birth weight (<2500 g) and a threefold risk of very low birth weight (VLBW; <1500 g) compared with White infants [1]. VLBW is a major contributor to neonatal mortality [2].

**Fig. 1:**
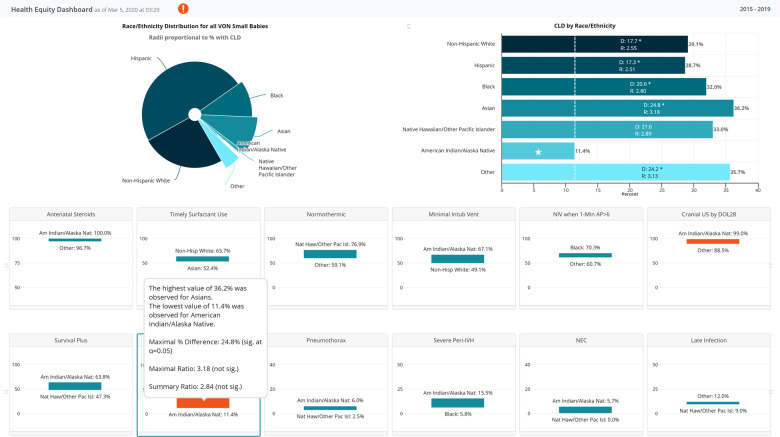
California Perinatal Quality Care Collaborative’s Very Low Birth Weight Infant Health Equity Dashboard. Each member of NICU can confidentially access a NICU, regional level equity report. The pie chart provides information on the racial/ethnic distribution of the population, the top row shows process measures, and the bottom row shows outcome measures. Statistically significant racial/ethnic differences between a top versus a bottom performing group are highlighted in orange. Selection of a measure (here, chronic lung disease (CLD)), shows a significant difference between Asian vs American Indian/Alaska natives. Selection also provides further detail in the bar chart on the upper right. The best performing racial/ethnic group (while reaching a minimal sample threshold) is indicated with a star. D: indicates the difference to the best performing group. R: indicates the ratio compared with the best performing group. Further detail can be explored by hovering over individual bars or by clicking on the table icon.

While traditionally, researchers have focused on the role of social determinants on health outcomes, recently, researchers have highlighted that neonatal intensive care unit (NICU) quality of care delivery may compound or reduce disparities by identifying those at higher risk [3]. Although overall outcomes and equities have improved [4], inequities in care delivery have been demonstrated to exist. Vulnerable populations are segregated into lower quality NICUs [5–9] and within NICUs they tend to receive lower quality of care, particularly less family-centered care [10]. Recent studies have created multifaceted activities centered around using quality improvement (QI) strategies to address equity in care delivery. Parker et al. conducted a statewide improvement collaborative addressing disparities in mother’s milk provision in Massachusetts [11]. The Vermont Oxford Network (VON) [12] has included equity relevant aims in their improvement collaboratives and recently announced the creation of a health equity network. At the California Perinatal Quality Care Collaborative (CPQCC) [13], a population-based statewide QI network, we have launched a health equity task force to address inequities in the care and outcomes of our patients [14]. Audit and feedback is foundational to QI activities, and the CPQCC has therefore recently introduced a health equity dashboard (Fig. 1), allowing individual NICUs to assess areas of concern within their own NICU.

While this initial iteration of an equity dashboard focuses on measures that had been associated with disparate care in the literature [3], a key missing ingredient for QI efforts in the NICU setting has been a lack of data on social factors including maternal social status, neighborhood, and NICU catchment area characteristics. Multilevel social factors data provide key contextual information on social determinants of health challenges families face prior, during and after their infant’s NICU stay. Previous studies have shown that neighborhood factors including racial residential segregation [15–17], income inequality [18], greenspace [19–21], socioeconomic status [22, 23], and built environment [24] are associated with birth outcomes. However, the pathways by which these factors may affect the quality of care delivery in the NICU are not well understood [25]. Social (e.g., neighborhood deprivation, which may affect mother’s advocacy role) and built environments (e.g., walkability, which may affect mother’s health) have been shown to affect pregnancy outcomes and mortality [26–28]. These factors are likely to be related to NICU quality through several pathways, including access to high quality NICUs, social resources influencing ability for mothers to advocate for self and child, other issues including language and transportation barriers, and need for coordinated local support after discharge. For example, neighborhood deprivation may limit infant breastfeeding rates due to knowledge levels, social support, work requirements, or stress [11].

Currently, NICU providers lack granular information about their populations’ neighborhood challenges, and their effects on care. To address this gap, the CPQCC plans to enrich the health equity dashboard with NICU catchment area data (i.e., census tract-level data highlighting elements that are modifiable). This paper provides background on the multilevel social factors considered for this effort, a description of the methods used to define NICU catchment areas, and an exploratory face validity check on the bivariate association of these measures with NICU quality of care. To study these relationships, we used a previously developed measure of NICU quality of care, the Baby-MONITOR, a composite indicator of nine risk-adjusted measures of quality [29, 30]. We hypothesized that social and built environment conditions in NICU’s catchment areas are associated with Baby-MONITOR scores.

## Materials and methods

### Study population

Clinical data were obtained from the CPQCC data registry, and included VLBW infant factors such as sex, gestational age, Apgar scores, location of birth, and maternal factors such as race/ethnicity, receipt of prenatal care, parity and mode of delivery. The CPQCC includes 134 NICUs and captures >95% of VLBW NICU admissions in California and is described in detail elsewhere [13, 31]. Briefly, the CPQCC maintains a demographically and biologically rich, real-time population-based database and unique links to several data sources, including California Children’s Services, Office of Vital Records, and the Office of Statewide Health Planning and Development. This linkage allowed us to access sociodemographic data such as maternal residence at birth, maternal education, payer source, and maternal country of birth.

For each NICU, maternal racial/ethnic composition and education composition measures were calculated as percentages of specific racial/ethnic groups (White, Black, Hispanic, and Asian/Pacific Islander) or education level (<high school, high school graduate, some college and associate degree, and bachelor’s degree and above).

For each NICU, we assessed percent of infants who were admitted at the nearest NICU, as determined by feature based proximity analyses in ArcMap (i.e., calculating straight line distance between maternal address and nearest NICU).

Maternal addresses at birth were geocoded using SAS 9.4 (PROC GEOCODE) to acquire latitude and longitude coordinates and assigned 2010 census tract identifiers to append neighborhood-level data on social and built environment attributes from the California Neighborhoods Data System [32]. Maternal addresses of 856 (0.5%) infants were not able to be geocoded. Several neighborhood-level factors were derived from the U.S. Census and the American Community Survey (2007–2011), including racial/ethnic composition, socioeconomic status (SES), population density, percent commuting by car/motorcycle, and household crowding [33, 34]. Neighborhood SES was based on a validated composite measure created by principal component analysis of data on education, housing cost, employment, occupation, income, and poverty; [35, 36] population density was measured as persons per km^2^ and commute patterns were measured as proportion of residents who commuted to work by car/motorcycle. Additional data on the built environment were obtained from NAVTEQ’s NAVSTREETS database including street connectivity and parks (per 1000 residents) [37]. Street connectivity was measured using the gamma index, a commonly used measure of walkability, and defined as the ratio of the actual number of street segments to the maximum possible number of intersections [38]. Business data were obtained from Walls & Associates’ National Establishment Time-Series Database from 1990 to 2008 [39] using a 3-year business activity window for 2006–2008 to capture businesses and recreational facilities (per 1000 residents), retail food environment index (unhealthy/healthy food outlet) [40, 41], and restaurant environment index (unhealthy/healthy restaurants) [42, 43]. These measures were categorized into quintiles based on their statewide distributions. Traffic counts data were obtained from the California Department of Transportation to measure traffic density [44, 45].

### NICU catchment area

Catchment areas were defined for each NICU by ranking census tracts in descending order based on the number of mothers/infants who lived within each tract. We selected census tracts that included at least 80% of the infants served by each NICU, giving priority to tracts that were nearer to the NICU in case of ties in the number of mothers/infants across census tracts served by a specific NICU.

**Fig. 2:**
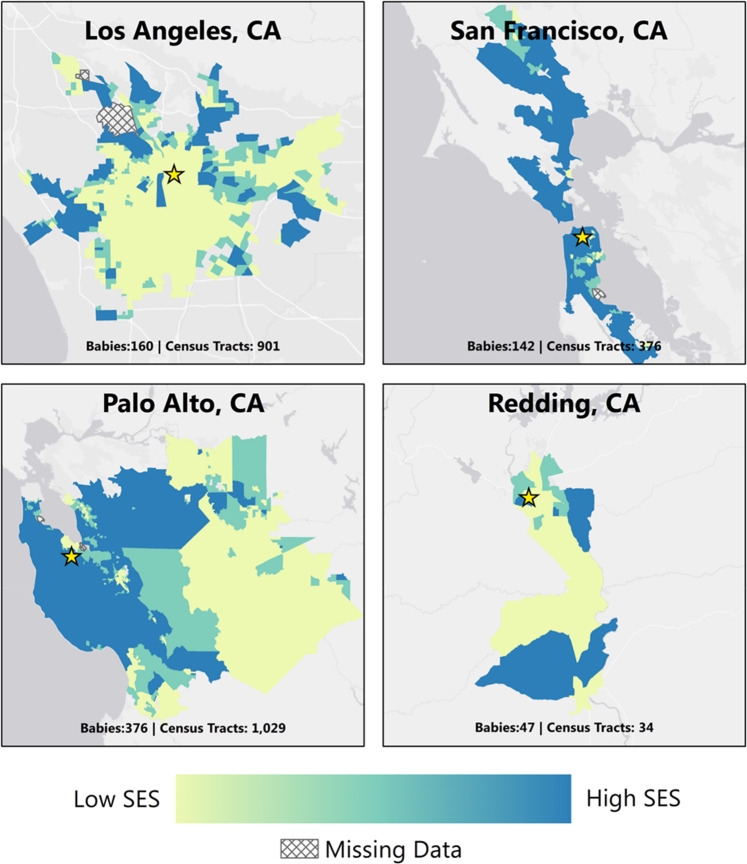
Maps of four NICU catchment areas from the California Perinatal Quality Care Collaborative. These maps for NICUs were chosen to illustrate the variability in catchment areas across NICUs in the Collaborative. The stars indicate the location of the NICU within their respective catchment areas. The colors of the census tracts within the catchment areas correspond to a neighborhood socioeconomic status (nSES), with the gradient of low (yellow) to high (blue) nSES based on statewide quintiles.

Census tracts without infants, but within the catchment area (i.e., surrounded by census tracts with mothers/infants) were included in the catchment to create a contiguous catchment area (Fig. 2). The catchment areas represented 7501 of the 8057 census tracts in CA; this included 1176 census tracts without infants. The NICU catchment areas varied in size with a median number of 228 census tracts per catchment area (IQR = 318). Many catchment areas overlapped (median 4, range 1–17). Once catchment areas were defined, we characterized multilevel social and built environment attributes for each area. We averaged the estimates among census tracts within each NICU catchment area. SES of catchment areas for sample NICUs are shown in Fig. 2.

### NICU quality

Quality of care was measured at the NICU level with the Baby-MONITOR score using NICU admission-level data, including transfers. The Baby-MONITOR score has previously been described in detail [9, 29, 30]. Briefly, Baby-MONITOR measures for the composite scale include (1) any antenatal steroid administration; (2) moderate hypothermia (<36 °C) on admission; (3) nonsurgically-induced pneumothorax; (4) hospital-acquired bacterial or fungal infection; (5) oxygen requirement at 36 weeks’ gestational age; (6) retinopathy of prematurity screening at the age recommended by the American Academy of Pediatrics; (7) discharge on any human milk; (8) mortality during the birth hospitalization; and (9) growth velocity calculated by using a logarithmic function. Individual components are risk adjusted and standardized against the California reference population of VLBW infants. An observed minus expected score is computed for each component, additively aggregated, and averaged. Scores below zero indicate worse than expected quality, scores above zero indicate better than expected quality, given a NICUs case mix.

### Statistical analyses

We examined NICU-level social and built environment factors and compared them across tertiles of NICU quality of care, as measured by Baby-MONITOR Score. At the NICU level, we describe both the NICU patient population and the NICU catchment area to compare these differences. The summary statistics included means and standard deviations or proportions by category. The statistical differences between each factor across Baby-MONITOR tertiles were determined by Pearson’s chi-squared tests for categorical factors and analysis of variance for continuous factors.

## Results

Infants who were cared for in member NICUs of the CPQCC and born between January 1, 2008 and December 31, 2011 were included in this study (*N* = 19,194). We restricted the cohort to admissions for infants who were between 401 and 1500 g or between 22 and 29 weeks gestational age at birth (*N* = 21,680) and did not die in the delivery room or before 12 h of life (*N* = 20,008) nor had severe congenital abnormalities associated with increased mortality risk (*N* = 17,871) [29]. We further excluded admissions missing maternal race/ethnicity for a final cohort of 17,781 admissions from 15,901 unique infants. The mothers were majority Hispanic with high school or less than high school education and Medi-Cal (CA’s Medicaid) health insurance. Their infants had a mean birth weight of 1059 g and were born at 28 weeks gestation; a quarter of births classified as small for gestational age (Table 1).

**Table 1:** Sample characteristics of very low birth weight infants in California NICUs, California Perinatal Quality Care Collaborative 2008–2011.

Infant and maternal characteristics	*N* (15,901) or mean	% or SD
*Infant factors*		
Birth weight in grams (mean, SD)	1070.62	284.4
Gestational age (mean, SD)	28.23	2.9
5 min Apgar Score (mean, SD)	7.53	1.8
Small for gestational age^a^		
No	11,584	72.9%
Yes	4314	27.1%
Outborn status		
No	12,326	77.5%
Yes	3575	22.5%
Sex^a^		
Female	7630	48.0%
Male	8270	52.0%
Singleton		
No	4341	27.3%
Yes	11,560	72.7%
NICU level Baby-MONITOR components		
Any antenatal corticosteroid administration (*N* = 11,443)	9871	86.3%
Moderate hypothermia (<36 °C) on admission (*N* = 15,695)	2348	15.0%
Nonsurgically-induced pneumothorax (*N* = 15,894)	591	3.7%
Health care-associated bacterial or fungal infection (*N* = 15,335)	1976	12.9%
Chronic lung disease (*N* = 13,593)	2925	21.5%
Timely eye exam (*N* = 10,604)	9940	93.7%
Discharge on any human breast milk (*N* = 13,776)	8855	64.3%
Mortality during the birth hospitalization (*N* = 15,638)	1068	6.8%
High growth velocity (*N* = 12,831)	6667	53.8%
*Maternal factors*		
Race/ethnicity		
NH White	4115	25.9%
NH Black	2162	13.6%
Hispanic	7594	47.8%
Asian/Pacific Islander	1630	10.3%
Other	400	2.5%
Maternal age^a^		
≤20	2204	13.9%
21–34	10,018	63.0%
35+	3664	23.0%
Nativity^a^		
Foreign born	5977	37.6%
US born	9900	62.3%
Education		
<High school	3787	23.8%
High school graduate	4160	26.2%
Some college and associate degree	3856	24.3%
Bachelor’s degree and above	3290	20.7%
Unknown	808	5.1%
Expected principal source of payment for delivery^a^		
Medi-Cal	7803	49.1%
Private insurance company	6994	44.0%
Other government programs	323	2.0%
Self pay	423	2.7%
Other	297	1.9%
Prenatal care^a^		
No	516	3.3%
Yes	15,282	96.1%
Cesarean delivery^a^		
No	4042	25.4%
Yes	11,858	74.6%
Cigarette use during pregnancy		
No	15,097	94.9%
Yes	510	3.2%
Unknown	294	1.9%
Parity^a^		
1	6663	41.90%
2	4295	27.01%
3+	4918	30.93%

^a^Missing data are not shown as <1.0%.

Descriptive statistics are provided for the subcomponents of the Baby-MONITOR (Table 1). The 119 NICUs in our cohort served a median of 101 babies from 2008–2011 (IQR = 112). With regard to NICU population characteristics, infants’ maternal racial/ethnic and educational composition of patient pools and NICU catchment areas, differed across tertiles of Baby-MONITOR scores (Table 2). NICUs in the lowest tertile of Baby-MONITOR scores had the largest populations of Black and Hispanic infants, and higher proportion of patients with education limited to high school or less. The percentage of infants cared for at the nearest hospital did not differ across Baby-MONITOR scores. With regard to NICU catchment area characteristics, NICUs caring for infants from higher neighborhood SES areas were more represented in the highest tertile of Baby-MONITOR scores. NICU quality also differed across several other neighborhood catchment area factors: parks per 1000 population, street connectivity, and proportion of population working from home were associated with higher NICU quality (*p* values < 0.1, Table 2). Higher levels of crowding in housing (defined as more than one person per room) and higher proportion of foreign-born composition were associated with lower NICU quality (*p* values < 0.1, Table 2).

**Table 2:** Bivariate associations of NICU characteristics with quality, Baby-MONITOR scores, in California 2008–2011 (*N* = 119 NICUs).

Characteristics	All		Baby-MONITOR score (tertile based on distribution of NICU)						*P* value (Chi-square test or ANOVA test)
			Tertile 1 (lowest quality)		Tertile 2		Tertile 3 (highest quality)		
	*N* or mean	% or SD	*N* (39) or mean	% or SD	*N* (40) or mean	% or SD	*N* (40) or mean	% or SD	
*NICU patient population characteristics*^a^									
Maternal racial/ethnic composition									
% NH White (mean, SD)	26%	15	20%	11	26%	14	32%	16	**<0.01**
% NH Black (mean, SD)	14%	11	16%	11	13%	11	12%	10	0.36
% Hispanic (mean, SD)	48%	17	53%	13	48%	18	44%	19	**0.03**
% Asian/Pacific Islander (mean, SD)	10%	9	8%	8	12%	12	10%	5	0.40
Maternal education composition									
% <High school (mean, SD)	24%	14	29%	13	21%	13	22%	13	**0.04**
% High school graduate (mean, SD)	26%	9	29%	8	26%	11	24%	8	0.86
% Some college and associate degree (mean, SD)	24%	8	24%	8	23%	9	26%	6	0.74
% Bachelor’s degree and above (mean, SD)	21%	15	14%	9	24%	18	23%	14	**0.02**
% of infants cared for at nearest NICU to maternal address	38%	29	37%	28	37%	30	41%	31	0.75
*NICU catchment area characteristics*^a,b^									
Tertile 1 of composite nSES score (lowest SES)	39	32.8%	21	53.9%	10	25.0%	8	20.0%	
Tertile 2 of composite nSES score	40	33.6%	12	30.8%	15	37.5%	13	32.5%	
Tertile 3 of composite nSES score (highest SES)	39	32.8%	6	15.4%	14	35.0%	19	47.5%	**0.01**
Total population (mean, SD)	47889	5945	4772	6423	4836	669	4758	468	0.83
Population density (mean, SD)	3628	2056	3891	2233	3948	2332	3061	1423	0.10
% NH White (mean, SD)	34%	15	30%	14	30%	15	41%	15	**<0.01**
% NH Black (mean, SD)	7%	6	9%	7	6%	5	6%	4	**0.03**
% Hispanic (mean, SD)	44%	16	48%	13	48%	17	37%	14	**<0.01**
% Asian/Pacific Islander (mean, SD)	12%	8	11%	6	14%	10	13%	8	0.28
% Foreign born (mean, SD)	30%	9	30%	9	33%	9	27%	8	**0.02**
% Crowding (mean, SD)	12%	6	13%	6	12%	6	9%	5	**0.01**
% Traveled to work by car/motorcycle (mean, SD)	85%	7	85%	6	85%	8	86%	5	0.83
% Traveled to work by public transport (mean, SD)	5%	5	6%	5	6%	6	5%	4	0.46
% Traveled to work by walk/bike (mean, SD)	1%	1	1%	0	1%	0	2%	1	0.34
% Work at home (mean, SD)	4%	1	4%	1	4%	1	5%	1	0.08
% Traveled 60+ min to work (mean, SD)	3%	1	3%	1	3%	2	3%	1	0.32
Street connectivity/gamma (mean, SD)	0.5	0	0.5	0	0.5	0.0	0.4	0.0	0.08
Traffic density (mean, SD)	100448	50218	100582	54776	108853	53303	92121	41618	0.34
Parks per 1000 population (mean, SD)	0.7	0.4	0.6	0.5	0.6	0.4	0.8	0.4	0.05
Total businesses per 1000 population (mean, SD)	160	408	223	674	112	98	145	200	0.47
Recreational facilities per 1000 population (mean, SD)	3.9	9.7	5.4	15.6	2.5	3.3	3.8	5.3	0.43
Restaurant Environment Index (REI) (mean, SD)	0.3	0.1	0.3	0.1	0.3	0.1	0.3	0.1	0.23
Retail Food Environment Index (RFEI) (mean, SD)	1.3	0.3	1.3	0.3	1.2	0.3	1.3	0.3	0.93

^a^The following variables did not have statistically significant distributions that varied by NICU quality tertiles: Hospital characteristics (Neonatologist available 24 h per day; type of hospital ownership; teaching hospital; AAP level of care; number of NICU beds; number of NICU admissions; registered nursing hours per patient day; number of licensed NICU beds; number of available NICU beds; number of staffed NICU beds), NICU patient characteristics (maternal racial/ethnic composition—% NH Black, % NH API; maternal education composition—% high school graduate, % some college/associate degree), catchment area characteristics (total population, population density, % API, % commute by car/motorcycle, public transportation, walk/bike, % work at home, traffic density, parks per 1000 population, total businesses, recreational facilities and food environment).

^b^Average of tract-level measures within each catchment area.

## Discussion

To better understand and address the contribution of multilevel social factors on inequities in NICU quality of care delivery, we examined associations with infant, maternal, and neighborhood factors in a population-based multilevel dataset. This multilevel compendium allows for a detailed examination of social factors by NICU patient population and catchment area and we provide initial insights into the associations between social factors and quality of care.

Catchment area specific neighborhood-level factors were associated with NICU Baby-MONITOR scores. Some of these may not be immediately modifiable by NICU providers, including patients residing in census tracts of lower neighborhood SES, higher population density, more household crowding, and resident compositions of more Black, Hispanic, and foreign-born residents. Nevertheless, these results provide face validity in highlighting the relation between residence in neighborhoods with more adverse social and built environment factors and lower NICU quality scores. Other associated factors may be addressable by NICU providers or hospital systems. For example, parks per capita, street connectivity, and higher proportion of the population working from home are marginally associated with higher NICU quality scores. While prima facie, these may seem beyond the reach of the NICU, providers could support mothers’ physical and emotional health constrained by limited access to parks by offering similar activities supporting family well-being on or near hospital grounds. Efforts to improve transportation for families to the hospital could be undertaken and remote viewing implemented to support parent bonding. Furthermore, with an increasing focus on population health and health equity, hospitals are encouraged to work with communities to address social factors that impact the health including addressing adverse neighborhood conditions. Hospitals are often the largest employers in a neighborhood, or city, and have the ability to contribute to more equitable economic development. Such efforts may improve financial viability through reduced readmissions and the development of a workforce that better matches the community with more opportunities to increase access to resources including quality health care.

This work adds to the literature examining the relationship of factors at various levels and NICU quality. In a study among NICUs in the VON, Black infants were concentrated at NICUs with lower quality scores, and Hispanic and Asian infants were at NICUs with higher quality scores, compared with White infants [8]. Racial/ethnic disparities in morbidity and mortality among very preterm infants in New York City were attributable to both infant factors and birth hospital implying hospital level factors may contribute to inequities in outcomes among NICU patients [46]. Conversely, maternal and neighborhood factors did not strongly influence NICU outcomes in the New York City study, though they are known risk factors to adverse birth outcomes including preterm birth [46]. Analyses of associations between zip-code level racial and economic segregation and preterm birth and infant mortality in California showed that women and infants in less privileged zip codes were at increased odds for these adverse outcomes [47]. The contributions of such multilevel resources have also been demonstrated in other areas of health; in particular, this is an emerging area of research in cancer epidemiologic studies [48, 49].

Our study has several strengths including combined data from a wide range of social factors at multiple levels. It uses a large, statewide, clinical, population-based database with a diverse study population with regard to race/ethnicity, SES, and geography. We also examined novel factors in relation to NICU quality such as small-area level neighborhood factors aggregated at the NICU catchment area level. Future efforts in this area are easy to envision. More detailed multilevel analyses will need to be conducted to address the independent and joint contributions of NICU catchment area-level neighborhood factors on clinical outcomes and quality of care. These multilevel factors could also be combined into a composite index with relevant NICU indicators such as quality or outcomes, assisting NICU providers in assessing population risk and policy makers in addressing inequities. Moreover, exploration of the need to add social factors into risk adjustment models for comparative assessment of NICU performance may be warranted. This is important because hospitals serving high-risk populations may require higher reimbursements to address incremental social needs, but may be disadvantaged in performance assessments that fail to assess social risk. Finally, health systems can use neighborhood-level data to understand and mitigate social risks. At the CPQCC, we are currently working to include neighborhood factors in NICU feedback reports along with their quality data with respect to outcomes and processes. Our hope is that NICUs will use this information to address barriers families face during and after the birth hospitalization as well as leverage community resources. Identifying neighborhood risk factors may help focus community resources to mitigate population risk for newborn infants.

Our study should be viewed in light of its design. We provide an initial assessment of associations between social factors and NICU quality of care delivery. While the analyses are adjusted for infant level characteristics, future analyses will need to examine the independent and joint roles of multilevel maternal, infant, hospital, and neighborhood factors in driving NICU inequities to further address confounding. Our results should thus be viewed as hypothesis generating. In addition, input data for this study are quite dated and will need to be updated with the next census survey in 2020. Finally, findings are restricted to California and generalizability to other states is unknown. In fact, a recent study highlighted the comparatively better outcomes for minority groups in California [8]. Most of the social factors in this study are derived from national data sets and could thus be replicated.

## Conclusion

We introduce a novel dataset of social and built environment factors and highlight associations between NICU patient population factors and multilevel social factors with NICU quality of care. NICU care providers will need to learn how to recognize, and ultimately help address, multilevel factors including social and built environment barriers facing their patients to facilitate better care and outcomes.
